# Worldwide Evaluation of CAMS-EGG4 CO_2_ Data Re-Analysis at the Surface Level

**DOI:** 10.3390/toxics10060331

**Published:** 2022-06-17

**Authors:** Danilo Custódio, Carlos Borrego, Hélder Relvas

**Affiliations:** 1 Helmholtz-Zentrum Hereon, Institute of Coastal Environmental Chemistry, Max-Planck-Str. 1, 21502 Geesthacht, Germany; 2 Max-Planck Institute for Biogeochemistry, 07747 Jena, Germany; 3 CESAM & Department of Environment and Planning, University of Aveiro, 3810-193 Aveiro, Portugal; cborrego@ua.pt (C.B.); helder.relvas@ua.pt (H.R.)

**Keywords:** compliance of CO_2_ re-analysis data, atmospheric CO_2_ trend, Copernicus Atmosphere Monitoring Service, uncertainties in CO_2_ data products, reducibility of anthropogenic fluxes

## Abstract

This study systematically examines the global uncertainties and biases in the carbon dioxide (CO_2_) mixing ratio provided by the Copernicus Atmosphere Monitoring Service (CAMS). The global greenhouse gas re-analysis (EGG4) data product from the European Centre for Medium-Range Weather Forecasts (ECMWF) was evaluated against ground-based in situ measurements from more than 160 of stations across the world. The evaluation shows that CO_2_ re-analysis can capture the general features in the tracer distributions, including the CO_2_ seasonal cycle and its strength at different latitudes, as well as the global CO_2_ trend. The emissions and natural fluxes of CO_2_ at the surface are evaluated on a wide range of scales, from diurnal to interannual. The results highlight re-analysis compliance, reproducing biogenic fluxes as well the observed CO_2_ patterns in remote environments. CAMS consistently reproduces observations at marine and remote regions with low CO_2_ fluxes and smooth variability. However, the model’s weaknesses were observed in continental areas, regions with complex sources, transport circulations and large CO_2_ fluxes. A strong variation in the accuracy and bias are displayed among those stations with different flux profiles, with the largest uncertainties in the continental regions with high CO_2_ anthropogenic fluxes. Displaying biased estimation and root-mean-square error (RMSE) ranging from values below one ppmv up to 70 ppmv, the results reveal a poor response from re-analysis to high CO_2_ mixing ratio, showing larger uncertainty of the product in the boundaries where the CAMS system misses solving sharp flux variability. The mismatch at regions with high fluxes of anthropogenic emission indicate large uncertainties in inventories and constrained physical parameterizations in the CO_2_ at boundary conditions. The current study provides a broad uncertainty assessment for the CAMS CO_2_ product worldwide, suggesting deficiencies and methods that can be used in the future to overcome failures and uncertainties in regional CO_2_ mixing ratio and flux estimates.

## 1. Introduction

More than ever, carbon dioxide (CO_2_) monitoring is at the front line of climate change mitigation. Taking the lead in climate transition and emission control has become imperative. Nonetheless, the capability of monitoring atmospheric CO_2_ by combining numerical models, in situ and remote sensing observations has rapidly evolved to improve our understanding of CO_2_ fluxes, sources and sinks [1,2].

The integration of earth observation data may become a critical tool in supporting policies aimed at reducing CO_2_ emissions, a key anthropogenic driver of climate change [2,3]. Human activities are directly responsible for most of the CO_2_ emissions, particularly due to the use of fossil fuel, with emissions particularly high in urban areas. With a lifetime of hundreds of years and well-known sources, CO_2_ essentially disperse after being emitted, and reliable data-product of such atmospheric trace gas can be critical in studies of air pollution dispersion and source apportionment of many atmospheric trace gases [4,5]. In addition, it can potentially provide a second option for evaluating national inventory [6].

Monitoring global CO_2_ emissions is highly desirable. To support and foster such a system initiative, the ECMWF Integrated Forecasting System (IFS) provides global gridded estimates of the atmospheric CO_2_ mixing ratios. In the global greenhouse gas re-analysis dataset (EGG4), the CO_2_ fluxes from natural and anthropogenic sources are modeled, even making use of a coarse time-resolution inventory (monthly). This provides CO_2_ fluxes in the atmosphere in a wide range of scales, from diurnal to inter-annual.

The Greenhouse Gas re-analysis dataset from Copernicus Atmosphere Monitoring Service, CAMS-EGG4, makes intense use of data assimilation. It combines high-resolution global models together with in situ CO_2_ concentration networks across the world, as well as satellite data. This results in a globally complete and consistent dataset using a model of the atmosphere based on the laws of physics and chemistry.

To make CAMS-EGG4 data more reliable in understanding sources, sinks and the transport of atmospheric CO_2_ from the surface into the troposphere around the globe, the reliability of this data product should be evaluated based on fiducial observation measurement comparison. Quantifying the quality of such data products by decomposing the inherent uncertainty components is a challenging and key component in product reliability and its use. The overall objective in validating and evaluating uncertainties of earth observation data products, such as those provided by CAMS, is to explicitly answer the question: How good is the evaluated dataset?

Hence, the study aims to explore the potential of CAMS to monitor and analyze CO_2_ concentrations at global scales. We attempt to compare the CAMS CO_2_ data at surface level with high accurate in situ surface data.

As a contribution to the evaluation of CAMS-EGG4 CO_2_ re-analysis data, the atmospheric concentration of CO_2_ provided by CAMS were compared with observational data gathered by monitoring ground stations. The reference data used to evaluate the performance of CO_2_ data from the CAMS re-analysis at surface level consist of high-resolution in situ observations obtained from extensive CO_2_ measurement programs, such as Global Atmosphere Watch (GAW), Amazon Tall Tower Observatory (ATTO) and the Global Monitoring Laboratory of the National Oceanic and Atmospheric Administration (NOAA).

A comprehensive validation report for the global CAMS CO_2_ re-analysis is reported by Ramonet et al. [7], based on airborne observation and 23 background stations worldwide. The aforementioned authors reported an increasing near-surface bias and regional uncertainties associated with vegetation model features representing biogenic fluxes. However, further evaluation of CAMS re-analysis seeking the main sources of errors is still needed to understand its product compliance and misfit. In addition, such a study can bring insights into the CO_2_ fluxes parametrization considered in the CAMS model.

The product strength and concerns were enhanced by understanding the differences among observations and the product and the spatial differences. In comprehensive statistics comparing measurement data from hundreds of stations, we assess the compliance of the re-analyses of CO_2_ on a global basis. Furthermore, we discuss the re-analyses and observation mismatches, exploiting the model constraints reproducing concentration at boundary regions and anthropogenic fluxes.

The paper is structured as follows: Section 2 describes the data and the methodology used. In Section 3, the main results are presented and analyzed. Section 4 is devoted to the conclusions.

## 2. Datasets and Methods

### 2.1. Datasets

To evaluate the compliance of the CAMS CO_2_ product, we selected 160 worldwide sites monitoring this trace gas. The following subsections briefly describe the datasets considered in this study.

#### 2.1.1. CAMS-EGG4

The Copernicus Atmosphere Monitoring Service (CAMS) implemented by the European Centre for Medium-Range Weather Forecasts (ECMWF) is one of the most advanced global atmospheric models simulating the state of the atmosphere with accuracy similar to what is theoretically possible using a 4D-Var method [8,9].

CAMS provides trace gas products such as greenhouse gases for downstream applications in early warning systems, environmental monitoring, health services and climate research [10].

Known as the Integrated Forecasting System, it is a component of the Copernicus European Earth observation program. It was originally developed through a series of Monitoring Atmospheric Composition and Climate (MACC) research projects (MACC I-II-III) that provide near-real-time and re-analysis systems with modelling and data assimilation of trace gas mixing ratios and aerosol concentrations. In this study, we used CO_2_ from CAMS global greenhouse gas re-analysis (EGG4) products obtained from the Copernicus platform. The CAMS-EGG4 CO_2_ product evaluated refers to re-analysis model level products downloaded in July 2021/September 2021 at the CAMS catalogue [11]. The re-analysis CO_2_ CAMS-EGG4 data are available at three-hour intervals (starting at 00:00 UTC) and on a regular latitude–longitude grid of 0.75° × 0.75°. This study used products retrieved at the model levels 60, 59, 58, 57, 56, 55, 54, 53 (10 m, 34 m, 71.89 m, 124.48 m, 195.85 m, 288.57 m, 404.74 m and 546.11 m) geometric altitude described at https://confluence.ecmwf.int/display/UDOC/L60+model+level+definitions (last access on 1 May 2022). As a global re-analysis grid, it can be interpolated to the desired location, and its accuracy is based on the error characteristics of the assimilated data.

The CAMS makes intense use of satellite assimilation; it also assimilates atmospheric observations from aircraft networks [12] and radiosondes [13]. CAMS-EGG4, currently covering the period 2003–2020, uses 4DVar data assimilation in CY47R1 of ECMWF’s Integrated Forecast System (IFS). This system is continuously improving through the addition of new features and new model versions. 

CAMS-EGG4 assimilates surface CO_2_ fluxes from the terrestrial biosphere directly into the IFS using the CTESSEL carbon module [14]. Other sources and sinks of CO_2_ are prescribed from different inventory sources and datasets. The CO_2_ fluxes are not directly updated by the observations assimilated, but an online flux correction scheme is applied to correct bias. Such correction is performed by comparing the modelled biogenic fluxes with a climatology of optimized fluxes. CAMS meteorological initial conditions come from the ECMWF operational analyses. The specific EGG4 model configuration is documented in the listed papers below:

- Emissions for CO_2_ are documented in Agustí-Panareda et al. [15] and Massart et al. [16];
- Bias correction for CO_2_ ecosystem fluxes based on the Biogenic Flux Adjustment Scheme is documented by Agustí-Panareda et al. [17];
- Mass fixer configuration for CO_2_ is documented by Agusti-Panareda et al. [18] and Diamantakis and Agusti-Panareda [19]. The full description can be assessed in CAMS-EGG4 data documentation [20].

#### 2.1.2. Ground-Based Observation

To evaluate the performance of CO_2_ provided by the CAMS model (EGG4), we used “ground truth” data from ground stations. We used continuous CO_2_ observations from more than 160 in situ monitoring stations established in worldwide monitoring programs.

Monitoring towers for ground-based measurements facilitate the measurement of greenhouses gases. We used data from the following: the Global Atmosphere Watch (GAW) program of the World Meteorological Organization (WMO); the National Oceanic and Atmospheric Administration (NOAA) in the United States, a scientific and regulatory agency; and the Amazon Tall Tower Observatory (ATTO). In addition to Tall Tower, seven other tower stations sampling at different altitudes were used in this study, providing observation data up to 457 m height (agl). The ground-based monitoring system provides the physical and chemical references of CO_2_ concentration to evaluate global earth observation data products. The GAW, NOAA and ATTO observing projects provide datasets and services to decision-makers and the public, as well as for scientific evaluation and forewarning of changes in the air composition that may have adverse effects on the environment [21]. The ground stations can provide handy insights in evaluating earth observation data products as they are spread worldwide and provide accurate high-resolution data (0.1 ppmv).

**Figure 1:**
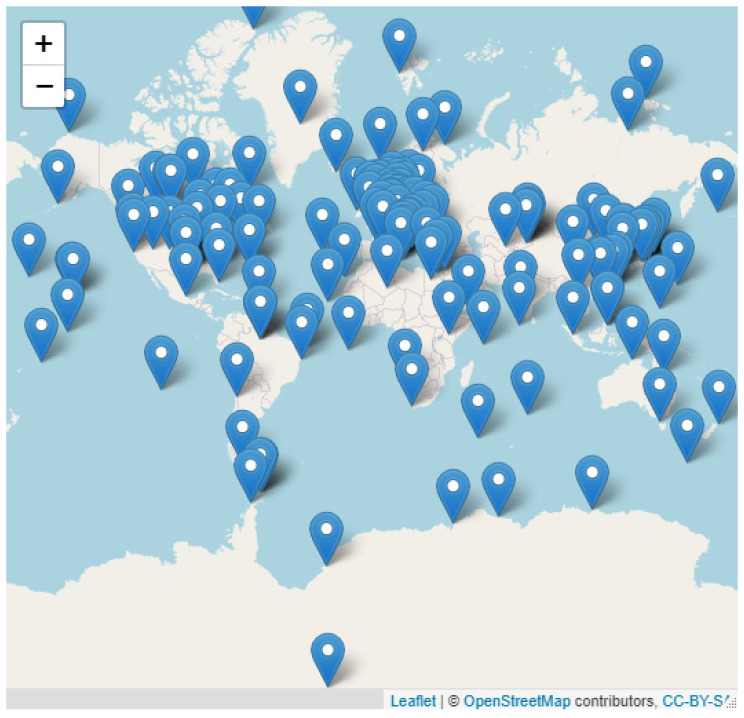
Location of stations considered in the evaluation of re-analysis CO_2_ provided by CAMS. The sta-tion name and its description can be accessed at the hypergraph link below (https://rpubs.com/danilocustodio/874276).

To estimate the accuracy of global CO_2_ re-analysis from CAMS, we selected the ground surface stations at different locations and altitudes. The selected sites are appropriate because they cover a long period of CAMS CO_2_ data availability, if not the entire period. The location of those stations (Figure 1), and the entire site description, are shown in the hypergraph at https://rpubs.com/danilocustodio/874276.

The CO_2_ ground observations considered at this study are fairly robust since they are derived from stable devices and a well-established validated method. Measurement uncertainties are reported by Andreae et al. [22], Stanley et al. [23], and GAW Report N° 255, [24].

### 2.2. Methods

For comparability with the re-analysis CO_2_ product, the observation data were averaged over three-hour periods. The observation data from ground stations at a 3 h resolution were considered valid only if they had values for at least 75% of the time.

The re-analysis (EGG4) products were downloaded at the regular latitude–longitude grid of 0.75° × 0.75° and then interpolated to observation data colocation. Despite the different spatial resolutions, the mean value of upper gridded earth observation products can be used to match up with ground monitoring sites [25,26].

Based on kernel-smoothing interpolation, the CO_2_ data products used in the comparison were interposed linearly in latitude, longitude and polynomially (second order) in atmospheric pressure to the same height as the measurement data.

After spatiotemporal collocation of CAMS and observation could be spatiotemporally aggregated and pairwise datasets were compared. The CAMS product and observations are compared, analyzed and discussed at the observation correspondent position. In this study, the temporal and spatial comparisons were performed for as long as it was conceivable and as broadly as possible, depending on the observation data availability.

After retrieving the CO_2_ product from the respective observation coordinates, temporal collocated pairwise datasets could be compared based on five metrics: The first metric is the mean difference between product values and observations, defined as root-mean-square error (RMSE) which shows the differences between product values and observation at different station sites. The RMSE is defined as (Equation (1)):

(1) $$\mathrm{RMSE}=\;\sqrt{\frac{1}{N}\sum_{n=1}^{n}{\left(X_{\mathrm{product}}-{\;X}_{\mathrm{observation}}\right)}^{2}{}^{\;}}\;\;\;$$

where N is the total number of observations, and X_product_ and X_observation_ are the CO_2_ mixing ratio for the product and observation, respectively. The second metric is relative differences (% difference) defined as the percentile difference of the product compared to the reference (Equation (2)).

(2) $$\%\;\mathrm{difference}=100\;\frac{1}{N}\sum_{n=1}^{n}\mathrm{ABS}\;\left(\frac{X_{\mathrm{product}}-{\;X}_{\mathrm{observation}}}{X_{\mathrm{observation}}}\;\right)$$

The third metric is mean bias (MB), or mean bias error (Equation (3)), which display the average bias in the CAMS-EGG4.

(3) $$MB=\frac{1}{N}\overset{n}{\underset{n=1}{\sum }}\left(X_{\mathrm{product}}-{\;X}_{\mathrm{observation}}\right)$$

The fourth adopted metric is the linear regression deployed to evaluate the conditional probability distribution of the product’s prediction (conditional quantile). Last but not least, kernelized temporal pattern is deployed to find and evaluate the temporal alignments among product and observation.

### 2.3. Representative and Spatial Scale Issues

Despite the different spatial resolutions, the mean value of models in an amplified grid cell is usually used to match up with ground monitoring sites [25,26].

However, it is worth highlighting two important issues when performing comparisons between gridded data such as those from CAMS, which have a large spatial coverage, and point-like ground-based observations: (i) a ground observing station might not be representative of the entire concentration over a grid cell, particularly in the case of a grid with intense sources, or in the vicinity of the sources; and (ii) the pixel-mean elevation of the surface (in gridded data) and the actual station elevation are not necessarily representative of the product layer.

These issues introduce errors and affect all comparative studies of this kind [27,28,29,30,31,32]. To mitigate this, the present study includes the comparison of aggregated factors of temporal pattern. In addition, we apply scale-height correction by interpolating the product dataset available at different layers to the height of the “ground truth” reference data. 

## 3. Results and Discussion

In this section, we present the main results from the evaluation of the carbon dioxide re-analysis data product provided by CAMS. To evaluate the compliance of CAMS-EGG4 compared to the observation, we apply mean bias, root-mean-square error (RMSE), relative differences, regression analysis and temporal patterns. Beyond the qualitative agreement and disagreement among the datasets, we assess the compliance strength of the CO_2_ product. Based on the five used metrics, we investigate the product’s misfit on the bias, response to temporal variation and uncertainties.

### 3.1. Overall Performance of Re-Analysis

**Figure 2:**
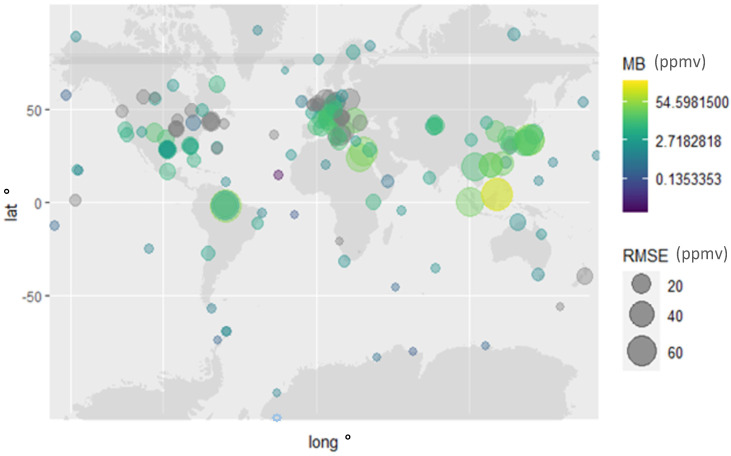
Mean bias (MB) and RMSE (in ppmv) displayed by CO_2_ re-analysis product provided by CAMS-EAC4 in the comparison with worldwide in situ observation considered in this study. The compliance metric for each station can be accessed through the hypergraph available at (https://rpubs.com/danilocustodio/874273).

The root-mean-square error (RMSE), average absolute errors in percentage, mean bias and slope regression between CAMS-EGG4 and observations are extracted from the entire available data and are presented at https://rpubs.com/danilocustodio/874273 (Figure 2). This hypergraph shows the data comparison between CAMS-EGG4 and observations at different sites considered in this study. The overall RMSE (C_observation_, C_CAMS-EGG4_) was 17.59 ppmv, the mean bias (C_observation_, C_CAMS-EGG4_) was 7.46 ppmv and the slope of the linear regression (C_observation_, C_CAMS-EGG4_) was 1.018.

The evaluation of the global performance of CO_2_ re-analysis from CAMS reveals a lower RMSE and a lower percentage of error at stations in remote environments, such as at the North and South Poles, in the Pacific, Atlantic and the Indian Oceans. On the other hand, higher disagreement among the compared datasets was mainly at stations in continental regions. 

The evaluated CO_2_ products were low biased and statistically similar to measurements (in 95% confidence interval in the mean) at the remote end stations, such as at the South Pole, King George Island, Samoa, Cape Verde, Azores, Barrow, Alert, Amsterdam Island, Macquarie Island, Ascension, Crozet, Mawson and Casey. At the aforementioned stations (georeferenced in https://rpubs.com/danilocustodio/874273), there is a difference of less than 0.5% between the CAMS data and the observations. On the other hand, differences rise above 6% at stations such as Amazon Tall Tower, Fundata, Monte Cimone, Bukit Kototabang, Pha Din, Mt. Dodaira, Suita, Kisai, Anmyeom-do and King’s Park. Notable differences were also observed at Farafra and Cairo stations; however, those stations have recently raised questions regarding their data quality control. 

**Figure 3:**
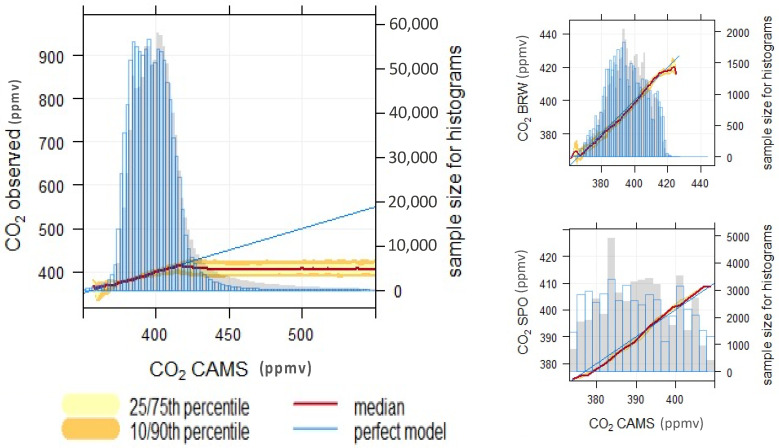
The conditional quantile regression plot shows the comparison between CAMS reanalysis and observation. The observations are split up into bins according to the corresponding CAMS value. On the left, the conditional quantiles of CAMS are displayed again worldwide ground-based observations, which include the data from all stations considered in this study. On the right, the regression specimen for Barrow in Alaska (top right) and South Pole (bottom right) are shown. The median prediction line together with the 25/75th and 10/90th quantile values are plotted together with a line showing a “perfect model”. Also presented is a histogram of CAMS values (shaded grey) and a histogram of observed values (shown as a blue line).

The minimum deviation of the CAMS data, less than 1.4 ppmv, was observed at Storhofdi (Iceland), Ascension Island (South Atlantic), Antarctic Station and Macquarie Island (Pacific Ocean), while the maximum deviation, above 50 ppmv, was observed at Danum Valley and Bukit Kotatabang (South Asia), Mt. Dodaria and Kisai (Japan). As shown in Figure 2, the CAMS-EGG4 data are in good agreement with CO_2_ observed at surface level in regions far from CO_2_ sources, indicated by a well-mixed ratio of this gas in the atmosphere. However, the CAMS product shows concentration dependence displaying significant bias at high CO_2_ concentrations (Figure 3). The average deviation between the CAMS data and observation is 2.4% (12.8 ± 15.5 ppmv). The reason CAMS overestimates CO_2_ in continental regions, as displayed in Figure 2, is likely related to different factors such as source consideration and transport modelling parametrization in the CAMS model. In addition, the Tall Tower data show that the differences are not vertically homogeneous; CAMS re-analysis data exhibit a strong gradient with improved performance at higher altitudes (Table S1, https://rpubs.com/danilocustodio/874273). The reasons for this could be attributed to the carbon sources and sinks at the near surface combined with atmospheric mixing, determining the spatial distribution and temporal variation of CO_2_ in the lower troposphere. The higher stratification of the atmospheric boundary can lead to large CO_2_ gradients that are more difficult for the re-analyses to accurately capture.

### 3.2. Compliance and Misfit

Figure 3 shows the conditional quantile plots where the re-analysis CO_2_ data are evaluated against the ground-based observations. The aforementioned figure illustrates the regression between re-analysis data derived from CAMS and observation at surface level for the worldwide stations described in Figure 1. The results indicate agreement among the dataset at more than 90% of the distribution. However, pronounced differences are displayed on the edges of the distribution when CAMS mainly overestimates CO_2_ concentrations and constrains bias above the 10/90th percentile. The CAMS data shows an increasing bias, up to 60 ppmv, at regions where potential nearby pollution sources are present. The misfit at the continental stations is probably due to the influence of CO_2_ fluxes close to the station, which are not spatially well resolved by the CAMS model, as is discussed in the next section.

As displayed at Barrow Station, CAMS could respond reasonably well at remote regions with low concentration variation in space. Displaying compliance in the 25/75th conditional quantile, the CAMS product could be an important reliable data source in remote ends or regions with a homogeneous distribution of CO_2_ mixing ratio.

**Figure 4:**
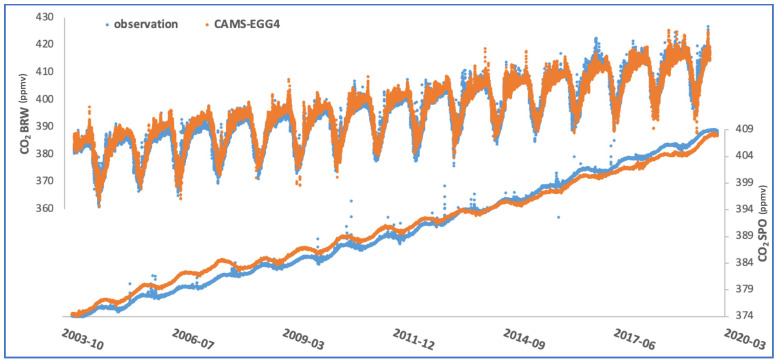
CO_2_ time series at Barrow (**top**) and South Pole (**bottom**) obtained from ground-based observa-tion and CAMS re-analysis. Both datasets are presented at a time resolution of 3 h.

As shown in Figure 4 for two specimen stations, the re-analysis matches the observation quite closely. However, the re-analysis shows a positive bias before 2013 and an increasing negative bias after that year. This bias and its signal break in 2013 are more easily detectable at stations in the southern hemisphere, such as at the South Pole, thanks to the weakness of the seasonal cycle. Still, it is present at other latitudes as is faintly displayed at Barrow. The bias inversion in 2012/2013 corresponds to the end of data assimilation from the SCIAMACHY instrument in 2012 and the start of assimilation of IASI-B data in 2013 [7].

**Figure 5:**
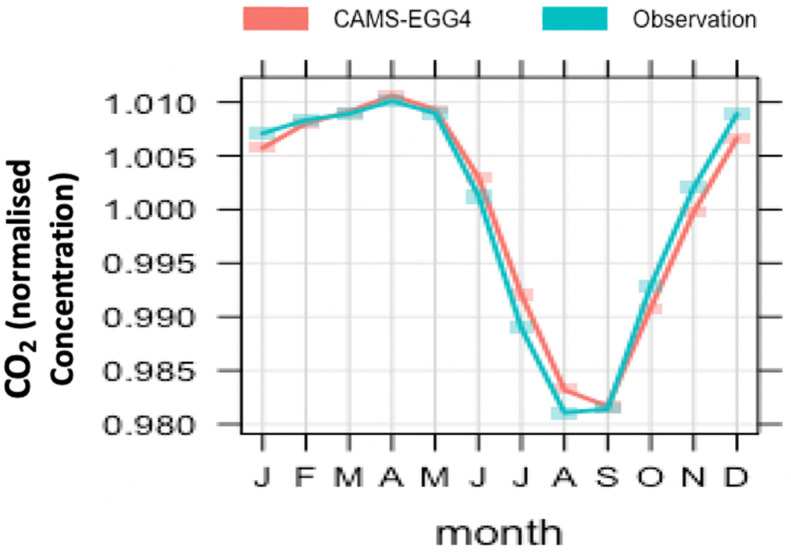
The annual CO_2_ cycle displayed by observation and CAMS includes all northern hemisphere sites considered at this study. The line represents the kernel average of the monthly concentration of CO_2_, and the shaded area represents its variance in a 95% significance interval.

The CAMS system reproduces the CO_2_ atmospheric background trend and seasonality fairly well. The performance of the re-analysis is compliant with the observed atmospheric CO_2_ trend in a 99% significance interval. The seasonal variability differs significantly from site to site. It is more pronounced in the northern hemisphere, which is explained by different biogenic fluxes, depending on the type of ecosystem (e.g., crops or forest areas) in the station footprint. The vegetation model in CAMS represents this well (Figure 5). 

We find that the temporal profiles of CO_2_ from CAMS re-analysis can agree exceptionally well with the CO_2_ temporal variability of ground-based observation at regions with low anthropogenic flux. The CAMS analysis appears to capture the biogenic fluxes in oceanic and terrestrial regions. For instance, a maximum diurnal variation of 1 ppmv was observed for oceans and terrestrial regions with a well-mixed CO_2_ ratio (background regions), with maxima at noon, when observed. Chen et al. [33] reported that the biogenic CO_2_ surface fluxes are provided by an online biosphere model in the IFS system. In addition, biases are corrected in real time throughout the forecast.

### 3.3. Main Sources of Error

Emissions and dynamics explain most of the differences in observation-data products comparisons [34]. 

Second Hedelius et al. [34] about 25% of the differences among CO_2_ data product and observation can be associated with the proximity of anthropogenic sources of CO_2_ combined with the basin topography that can lead to the trapping of polluted air enhancing CO_2_ concentration gradient within the basin. Such events are not solved by atmospheric models. This error is mainly associated with errors in the estimation of mixed layer height, leading to significant errors in retrieved fluxes. Kort et al. [35] and Newman et al. [36] attributed this phenomenon almost entirely to anthropogenic emissions.

As reported by Lian et al. [37] sources of misfits include uncertainties in the atmospheric transport, in atmospheric CO_2_ conditions that are used at the boundaries of the model, in the natural CO_2_ fluxes within the modeling domain, and in the spatial and temporal resolution and distribution of the inventories used in the model.

Uncertainties in modeling the atmospheric transport of CO_2_ are exacerbated in urban areas due to building obstacles (roughness) that generate specific mixing processes and modify the wind speed and direction [37]. This also occurs in misestimation of urban biogenic fluxes [38].

The uncertainties in the assumed temporal and spatial emission variations induce a critical source of error poorly constrained by the inversions due to the lack of boundary data [39,40]. The CAMS anthropogenic emission inventory’s spatial and temporal allocation has a monthly spatial grid resolution of 0.1° x 0.1°. The inventory accounts for emissions from different sectors, such as the transportation, residential and energy sectors, as well as biogenic fluxes [41]. Potential sources of CO_2_ not far from a station can cause variations in atmospheric CO_2_ that are not captured by the model grid cell, which are resolved in kilometric scale. 

Uncertainties in atmospheric transport of CO_2_ are exacerbated in areas near sources where mixing processes and variation in wind speed and direction can significantly take place. 

The results presented in this article show systematic biases in the re-analyses at mixing ratios above 420 ppmv. Further examination shows that most CAMS biases and high RMSE are located in regions with intense anthropogenic sources, especially in Asia. To analyze the errors constrained by uncertainty in anthropogenic fluxes, we evaluated the diurnal pattern of observation and re-analysis at the different stations, such as Kisai, Mt. Dodaria, Mikawa-Ichimomiyia and Suita in Japan, as well at stations in South Asia and Europe, where the re-analysis displayed high miss estimation. Those stations have the highest CO_2_ mixing ratios and are within or in the proximity of urban areas, potentially providing strong signatures of anthropogenic emissions.

**Figure 6:**
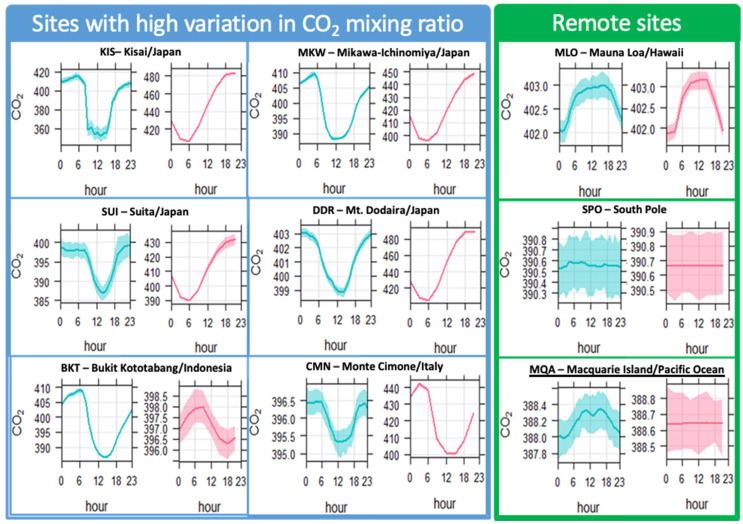
Diurnal cycle of CO_2_ (in ppmv) displayed by the observation (light blue) and CAMS (red). The blue panel shows the diurnal cycle at six stations with high CO_2_ mixing ratios variation; toward the left of the green panel, the diurnal variation at three remote stations with low CO_2_ fluxes is shown. The smooth line represents the kernel average of the diurnal concentration of CO_2_, and the shaded area represents its variance in a 95% significance interval.

Figure 6 shows the diurnal variation of CO_2_ at selected sites with high RMSE. For instance, the observation shows a flux of CO_2_ during the day. The most common pattern of diurnal variation displays a decrease of CO_2_ mixing ratio at noon. This could be linked to biogenic uptake and the sensible heat emissions at the surface that enhance vertical mixing in urban environments with the increase in depth of the boundary layer (Lian et al., 2021). 

Regions with high CO_2_ mixing ratio, such as urban and suburban areas, have green spaces and are surrounded by rural areas that can actively take up CO_2_ in the daytime during the growing season. 

Parametrization of CO_2_ fluxes misestimating the daytime biogenic respiration is a significant source of modeling error during the growing season outside the city or in regions with significant vegetation cover. The CO_2_ concentration is also influenced by remote emissions and large-scale biogenic fluxes in incoming air masses when the wind blows from green areas into urban environments [42].

The re-analysis shows a diurnal cycle amplitude quite different from the corresponding observation at stations such as Mt. Dodoira, Kisai, Mikawa-Ichinomiya and Suita. The most striking error is the concentration amplitude throughout the day when the observation shows a variation in CO_2_, while the re-analysis misestimates such fluxes. 

The impact of anthropogenic fluxes in continental conditions of high CO_2_ concentration, where the re-analysis performance is relatively small, would be associated with the signature of the anthropogenic emissions constrained in the IFS model, which are not observed in reality.

Since CAMS assimilates only infrequent remote sensing XCO_2_ measurements from the polar-orbiting satellite [32], the observations are likely insufficient to correct this bias. In addition, part of the bias may be inherited from biases in the satellite retrievals and their boundary limitation [16,43].

The good representation of annual cycle and the decrease in flux emissions during the daytime and an increase during the nighttime, in almost all stations with well-mixed CO_2_ ratio, is a strong indication that the seasonal and diurnal profile of the biogenic CO_2_ emissions is well represented in the IFS model in remote regions. On the other hand, the amplitude in the diurnal profile at regions with high CO_2_ mixing ratios could indicate an erroneous CO_2_ emission/sink in the CAMS inventory, and a flux parametrization, as an improved diurnal pattern, is needed. 

Errors in atmospheric transport also contribute to the re-analysis data misfit. The overestimation of the daytime CO_2_ mixing ratio, not only at the urban sites, but also at rural stations, is evidence of the flux parametrization deficiency. This mismatched pattern in the fluxes can embed a difference in the re-analysis of up to 80 ppmv that can explain most of the RMSE from the CO_2_ re-analysis. The IFS system may overcome these errors related to CO_2_ fluxes in boundary conditions by improving anthropogenic fluxes parametrization in urban/suburban/rural regions, as well as improving biogenic fluxes parametrization in those regions that can be significantly constrained by vegetation uptake or influenced by the incoming air masses from regions with large-scale biogenic fluxes. It may be addressed by assimilating CO_2_ gradients in upwind–downwind background stations rather than assimilating absolute CO_2_ concentrations.

## 4. Conclusions

This study examines biases and uncertainties in the CO_2_ mixing ratio from re-analysis CAMS-EGG4 data evaluated against hundreds of stations’ data distributed worldwide that provide long-term series of ground-based in situ measurements.

It has been found that the CAMS CO_2_ re-analyses agree reasonably well, into the 10/90th percentile, with the in situ measurements in regions with homogeneous CO_2_ distribution or in well-mixed air mass areas, and there is also a tendency to underestimate the observations. On the other hand, CAMS re-analyses exhibit some systematic positive biases at regions with high CO_2_ anthropogenic fluxes, which infer a mean bias (C_observation_, C_CAMS-EGG4_) in 7.46 ppmv, especially in Asia.

The CAMS product can realistically capture the upward trend in atmospheric CO_2_ as well as the biogenic CO_2_ fluxes throughout the seasons.

The results from towers show a substantial increase of uncertainty close to the surface. The CAMS uncertainties tend to decrease at lower product levels in the lowermost troposphere.

CAMS tends to overestimate the CO_2_ mixing ratio at regions with high CO_2_ concentration and underestimates it at regions with low fluxes.

An important finding from the evaluation of CO_2_ re-analyses is the low performance by CAMS-EGG4 in reproducing fluxes in urban and surrounding areas. This finding implies a limited spatiotemporal coverage of the CAMS system due to limited boundary conditions and limited resolution in the emission inventory used by the IFS model.

The current study provides an essential assessment of uncertainties in the fluxes of the CO_2_ mixing ratio. In addition to better spatial coverage, the results highlight the necessary improvement in diurnal parametrization and atmospheric transport through more refined regional modeling and boundary consideration for CO_2_ flux estimation.

The results also reveal that the estimation of re-analysis uncertainties using the differences between data product and ground-based observation can be valuable as a baseline reference. This information can be used for future improvement concerning the quantification of regional-scale uncertainties in CO_2_ mixing ratio and constraints in flux estimates by the ECMWF-IFS.

## Data Availability

The EGG4-CO_2_ data used in this study are available at Copernicus Atmosphere Monitoring Service (CAMS) Atmosphere Data Store (ADS) (https://ads.atmosphere.copernicus.eu/cdsapp#!/dataset/cams-global-ghg-reanalysis-egg4?tab=form, last access 1 May 2022); and the in-situ observation data used as a reference in the evaluation of EGG4-CO_2_ are publicly available at links provided throughout the article.

## Supplementary Materials

The following supporting information can be downloaded at: https://www.mdpi.com/article/10.3390/toxics10060331/s1, Table S1: Location, root-mean-squares error (RMSE), percentile (%) differences, mean bias (MB), and the slope of the regression between observation and CAMS-EGG4 at the studied stations. * The number subscripted on the acronym of the station refer to the height (m agl) of the observation in tower stations.
