# Supplementary material for: Worldwide Evaluation of CAMS-EGG4 CO2 Data Re-Analysis at the Surface Level
Source: Toxics. 2022 Jun 17;10(6):331. doi: 10.3390/toxics10060331 (PMC9229668; doi:10.3390/toxics10060331)
Supplement: Supplementary file 1 [file toxics-10-00331-s001.zip › toxics-1761906-supplementary.pdf]

toxics

MDPI

# Supplementary Materials: Worldwide Evaluation of CAMS-EGG4 $CO_2$ Data Re-Analysis at the Surface Level

**Danilo Custódio, Carlos Borrego and Hélder Relvas**

**Table S1.** Location, root-mean-squares error (RMSE), percentile (%) differences, mean bias (MB), and the slope of the regression between observation and CAMS-EGG4 at the studied stations. * The number subscripted on the acronym of the station refer to the height (m agl) of the observation in tower stations.

| Station | Site | latitude | longitude | RMSE | % difference | MB | slope |
|---|---|---|---|---|---|---|---|
| ALT | Alert (ALT, Canada) | 82.49915 | −62.3415 | 2.21 | 0.47 | −0.57 | 0.999 |
| AMS | Amsterdam Island (AMS, France) | −37.7983 | 77.5378 | 1.68 | 0.41 | 1.58 | 1.004 |
| ATTO $_{10m}$ | Amazon Tall Tower Observatory (ATTO $_{10\,m}$, Brazil) | −2.1441 | −58.9999 | 74.30 | 12.30 | 44.53 | 1.12 |
| ATTO $_{34m}$ | Amazon Tall Tower Observatory (ATTO $_{34\,m}$, Brazil) | −2.1441 | −58.9999 | 33.01 | 5.63 | 17.39 | 1.04 |
| ATTO $_{71m}$ | Amazon Tall Tower Observatory (ATTO $_{71\,m}$, Brazil) | −2.1441 | −58.9999 | 55.46 | 8.36 | 3.83 | 1.01 |
| AZR | Serreta (AZR,Terceira - Azores) | 38.77 | −27.38 | 1.85 | 0.40 | −0.33 | 0.999 |
| AMY | Anmyeon-do (AMY, Republic of Korea) | 36.5386 | 126.33 | 35.20 | 4.94 | 19.16 | 1.045 |
| BEO | BEO Moussala (BEO, Bulgaria) | 42.1792 | 23.5856 | 50.56 | | 63.11 | 1.164 |
| BHD | Baring Head (BHD, New Zealand) | −41.4082 | 174.8708 | 10.31 | 1.76 | −6.24 | 0.985 |
| BKT | Bukit Kototabang (BKT, Indonesia) | −0.20194 | 100.3181 | 52.71 | 9.72 | 30.69 | 1.074 |
| BRW | Barrow (BRW, Alaska) | 71.32301 | −156.611 | 2.77 | 0.54 | 1.22 | 1.003 |
| CDL | Candle Lake (CDL, Canada) | 53.98711 | −105.118 | 5.81 | 1.09 | 2.59 | 1.007 |
| CGO | Cape Grim (CGO, Australia) | −40.6822 | 144.6883 | 4.86 | 0.78 | 1.10 | 1.003 |
| CGR | Capo Granitola (CDR, Italy) | 37.57111 | 12.65972 | 8.30 | 1.35 | −2.46 | 0.994 |
| CLH | Churchill (CLH, Canada) | 58.7379 | −93.8206 | 3.89 | 0.63 | 1.97 | 1.005 |
| CMN | Monte Cimone (CMN, Italy) | 44.16667 | 10.68333 | 33.31 | 6.22 | 24.28 | 1.061 |
| CPA | Cholpon-Ata (CPA, Kyrgyzstan) | 42.63694 | 77.0675 | 13.35 | 2.12 | 5.17 | 1.012 |
| CPT | Cape Point (CPT,South Africa) | −34.3535 | 18.48968 | 3.86 | 0.61 | 1.64 | 1.004 |
| CUR | Monte Curcio (CUR, Italy) | 39.31597 | 16.42325 | 13.02 | 3.03 | 11.89 | 1.029 |
| CVO | Cape Verde (CVO) | 16.86403 | −24.8675 | 1.67 | 0.33 | 0.01 | 1.000 |
| DDR | Mt. Dodaira (DDR, Jajan) | 36 | 139.18 | 65.72 | 11.48 | 44.79 | 1.111 |
| DEU | Deuselbach (DEU, German) | 49.76667 | 7.05 | 22.86 | 3.46 | 11.41 | 1.029 |
| DIG | Diabla Gora (DIG, Poland) | 54.15 | 22.06667 | 21.91 | 3.66 | −7.93 | 0.978 |
| DMV | Danum Valley (DMV, Malaysia) | 4.981389 | 117.8436 | 75.24 | 14.41 | 50.67 | 1.129 |
| ECO | Lecce Environmental-Climate Observatory (ECO, Italy) | 40.3358 | 18.1245 | 28.47 | 3.52 | −12.56 | 0.967 |
| EGB | Egbert (EGB, Canada) | 44.23101 | −79.7838 | 8.15 | 1.16 | 0.32 | 1.001 |
| ESP | Estevan Point (SEP, Canada) | 49.38294 | −126.544 | 5.53 | 0.91 | −0.22 | 0.999 |
| ETL | East Trout Lake (ETL, Canada) | 54.35374 | −104.987 | 2.51 | 0.43 | −0.28 | 0.999 |

| | | | | | | | |
|---|---|---|---|---|---|---|---|
| **FDT** | Fundata (FDT, Romania) | 45.43147 | 25.27154 | 33.30 | 7.88 | 28.06 | 1.077 |
| **FRF** | Farafra (FRF, Egypt) | 27.05812 | 27.99016 | 49.95 | 11.47 | 36.06 | 1.088 |
| **FSD** | Fraserdale (FSD, Canada) | 49.87517 | −81.5698 | 6.33 | 0.84 | −1.05 | 0.997 |
| **GAT** | Gartow (GAT, Germany | 53.06551 | 11.44273 | 8.05 | 1.17 | 2.77 | 1.007 |
| **GLH** | Giordan Lighthouse (GLH, Malta) | 36.0722 | 14.2184 | 15.13 | 3.63 | 14.18 | 1.036 |
| **GSN** | Gosan (GSN, Republic of Korea) | 33.29382 | 126.1628 | 7.13 | 1.35 | −0.03 | 0.99 |
| **HPB** | Hohenpeissenberg (HPB, Germany) | 47.8015 | 11.00962 | 15.50 | 2.19 | 6.45 | 1.016 |
| **IZO** | Izaña (IZO, Tenerife) | 28.309 | −16.4994 | 3.01 | 0.61 | 1.90 | 1.005 |
| **JBN** | Jubany (JBN, Argentina) | −62.2382 | −58.666 | 1.60 | 0.33 | 0.84 | 1.002 |
| **JFJ** | Jungfraujoch (JFJ, Switzerland) | 46.54749 | 7.98509 | 23.13 | 4.18 | 15.42 | 1.040 |
| **JGS** | Jeju Gosan (JFJ, Republic of Korea) | 33.30005 | 126.2057 | 12.57 | 2.78 | 10.68 | 1.027 |
| **KIS** | Kisai (KIS, Japan) | 36.08 | 139.55 | 56.21 | 9.69 | 28.82 | 1.067 |
| **KIT** | Karlsruhe (KIT, Germany) | 49.1 | 8.438 | 21.08 | 2.98 | 5.44 | 1.013 |
| **KMW** | Kollumerwaard (KMW, Netherlands) | 53.33333 | 6.266667 | 25.08 | 3.82 | −13.47 | 0.964 |
| **KSG** | King Sejong (KSG, Republic of Korea) | −62.2167 | −58.7833 | 1.64 | 0.40 | 1.50 | 1.004 |
| **LIN** | Lindenberg (LIN, Germany) | 52.21667 | 14.11667 | 9.62 | 1.34 | 3.46 | 1.008 |
| **LLB** | Lac La Biche (LLB, Canada) | 54.95381 | −112.467 | 6.36 | 0.98 | −2.61 | 0.994 |
| **LMP** | Lampedusa (LMP, Italy) | 35.5182 | 12.6305 | 1.81 | 0.36 | −1.24 | 0.997 |
| **LMT** | Lamezia Terme (LMT, Italy) | 38.8763 | 16.2322 | 23.60 | 3.10 | −12.27 | 0.968 |
| **MEX** | Mexico, (MEX) | 18.9841 | −97.311 | 12.03 | 1.90 | 8.14 | 1.02 |
| **MHD** | Mace Head (MHD, Ireland) | 53.32661 | −9.90442 | 4.01 | 0.74 | 0.66 | 1.002 |
| **MKW** | Mikawa-Ichinomiya (MKW, Japan) | 34.85 | 137.43 | 39.89 | 7.22 | 19.73 | 1.048 |
| **MLO** | Mauna Loa (MLO, USA) | 19.53623 | −155.576 | 2.89 | 0.62 | 2.23 | 1.006 |
| **MNM** | Minamitorishima (MNM, Japan) | 24.2883 | 153.9833 | 1.86 | 0.39 | 0.97 | 1.002 |
| **MQA** | Macquarie Island (MQA, Australia) | −54.4985 | 158.9385 | 1.42 | 0.33 | −1.34 | 0.997 |
| **NGL** | Neuglobsow (NGL, Germany) | 53.14278 | 13.03333 | 15.36 | 2.18 | −6.38 | 0.983 |
| **PAL** | Pallas (PAL, Finland) | 67.97361 | 24.11583 | 6.35 | 1.18 | 2.53 | 1.006 |
| **PDI** | Pha Din (PDI, Viet Nam) | 21.5731 | 103.5157 | 49.19 | 8.40 | 13.28 | 1.032 |
| **PRS** | Plateau Rosa (PRS, Italy) | 45.93534 | 7.70731 | 22.39 | 4.24 | 16.28 | 1.041 |
| **PUY** | Puy de Dôme (PUY, France) | 45.7723 | 2.9658 | 14.48 | 2.38 | 8.42 | 1.021 |
| **RYO** | Ryori (RYO, Japan) | 39.0319 | 141.8222 | 18.63 | 2.92 | 9.74 | 1.024 |
| **SMO** | Samoa (SMO, USA) | −14.247 | −170.564 | 1.65 | 0.35 | 0.28 | 1.001 |
| **SNB** | SONNBLICK (SNB, Austria) | 12.95778 | 47.05417 | 4.45 | 0.86 | 0.42 | 1.001 |
| **SPO** | South Pole | −89.98 | −24.8 | 1.42 | 0.33 | 0.46 | 1.001 |
| **SSL** | Schauinsland (SSL, Germany) | 47.9 | 7.916667 | 25.62 | 4.61 | 17.44 | 1.044 |
| **SUI** | Suita (SUI, Japan) | 34.82 | 135.52 | 37.89 | 6.28 | 15.44 | 1.036 |
| **TIK** | Tiksi (TIK, Russian Federation) | 71.5965 | 128.8887 | 4.45 | 0.70 | 1.30 | 1.003 |
| **TKY** | Takayama (TKY, Japan) | 36.14617 | 137.4231 | 33.99 | 6.03 | 21.88 | 1.056 |
| **TLL** | El Tololo (TLL, Chile) | −30.1683 | −70.8036 | 6.13 | 1.12 | 3.43 | 1.009 |
| **TOH** | Torfhaus (TOH, Germany) | 51.8088 | 10.535 | 15.25 | 2.20 | 8.26 | 1.02 |
| **WES** | Westerland (WES, Germany) | 54.92314 | 8.308017 | 6.45 | 1.10 | −0.11 | 0.999 |

| | | | | | | | |
|---|---|---|---|---|---|---|---|
| **WSA** | Sable Island (WSA, Canada) | 43.93227 | −60.0126 | 3.01 | 0.58 | −1.68 | 0.996 |
| **YON** | Yonagunijima (YON, Japan) | 24.4667 | 123.0106 | 3.49 | 0.64 | 0.76 | 1.002 |
| **ZEP** | Zeppelin Mountain (ZEP, Norway) | 78.90669 | 11.88934 | 3.01 | 0.63 | 1.89 | 1.005 |
| **ZSF** | Zugspitze-Schneefernerhaus (ZSF, Germany) | 47.4165 | 10.97964 | 22.25 | 3.99 | 14.70 | 1.04 |
| **ABP** | Arembepe (ABP,Brazil) | −12.7667 | −38.1667 | 3.63 | 0.92 | 3.54 | 1.009 |
| **AMT 12m** | Argyle, Maine, United States (AMT 12m) | 45.0345 | −68.6821 | 18.21 | 2.32 | −5.16 | 0.986 |
| **AMT 30m** | Argyle, Maine, United States (AMT 30m) | 45.0345 | −68.6821 | 13.35 | 1.89 | −3.11 | 0.992 |
| **AMT 107m** | Argyle, Maine, United States (AMT 107m) | 45.0345 | −68.6821 | 9.46 | 1.54 | −1.35 | 0.996 |
| **ASC** | Ascension Island (ASC,United Kingdom of Great Britain and Northern Ireland) | −7.97 | −14.4 | 1.29 | 0.28 | 0.16 | 1 |
| **ASK** | Assekrem (ASK,Algeria) | 23.26667 | 5.633333 | 1.89 | 0.41 | 1.02 | 1.003 |
| **BAL** | Baltic Sea (BAL,Poland) | 55.5 | 16.67 | 3.54 | 0.74 | 1.54 | 1.004 |
| **BGU** | Begur (BGU,Spain) | 41.97 | 3.23 | 11.33 | 2.80 | 10.87 | 1.028 |
| **BME** | St. David's Head (BME,United Kingdom of Great Britain and Northern Ireland) | 32.37 | −64.65 | 3.59 | 0.74 | 2.01 | 1.003 |
| **BMW** | Tudor Hill (Bermuda) (BMW,United Kingdom of Great Britain and Northern Ireland) | 32.27 | −64.88 | 4.21 | 0.80 | −0.44 | 0.999 |
| **BSC** | Constanta (Black Sea) (BSC,Romania) | 44.17 | 28.68 | 8.067 | 1.49 | −5.08 | 0.987 |
| **CAI** | Cairo (CAI,Egypt) | 30.08333 | 31.28333 | 52.25 | 12.87 | 36.74 | 0.912 |
| **CBA** | Cold Bay (AK) (CBA,United States of America) | 55.2 | −162.717 | 2.44 | 0.49 | 0.22 | 1 |
| **CFA** | Cape Ferguson (CFA,Australia) | −19.2773 | 147.0584 | 2.33 | 0.49 | 1.60 | 0.969 |
| **CHM** | Chibougamau (CHM,Canada) | 49.69251 | −74.3423 | 4.44 | 0.94 | 3.38 | 0.992 |
| **CHR** | Christmas Island (CHR,Kiribati) | 1.7 | −157.17 | 3.98 | 0.66 | −1.24 | 0.853 |
| **CRI** | Cape Rama (CRI,India) | 15.08 | 73.83 | 5.61 | 0.98 | 3.77 | 1.01 |
| **CRZ** | Crozet (CRZ,France) | −46.4333 | 51.83358 | 1.35 | 0.31 | 0.29 | 1.001 |
| **CYA** | Casey (CYA,Australia) | −66.2833 | 110.5167 | 1.31 | 0.30 | 0.28 | 1.001 |
| **DMV** | Danum Valley (DMV, Malaysia) | 4.981389 | 117.8436 | 71.14 | 13.77 | 291.64 | 1.12 |
| **DRP** | Drake Passage (DRP,United States of America) | 59.00002 | −64.69 | 10.02 | 2.30 | 7.62 | 1.019 |
| **EIC** | Easter Island (EIC,Chile) | −27.1667 | −109.417 | 1.87 | 0.91 | 0.91 | 1.002 |
| **FKL** | Finokalia (FKL,Greece) | 35.3378 | 25.6694 | 2.89 | 0.65 | 2.20 | 1.005 |
| **GMI** | Guam (Mariana Island) (GMI,United States of America) | 13.43 | 144.78 | 1.88 | 0.42 | 1.08 | 1.003 |
| **GPA** | Gunn Point (GPA, Australia) | −12.2488 | 131.0453 | 10.42 | 3.31 | 1.40 | 1.021 |
| **HBA** | Halley (HBA,United Kingdom of Great Britain and Northern Ireland) | −75.5715 | −25.5039 | 1.45 | 0.34 | 0.93 | 0.974 |
| **HFD** | Heathfield (HFD,United Kingdom of Great Britain and Northern Ireland) | 50.97667 | 0.230556 | 2.82 | 0.54 | 0.98 | 1.002 |

| | | | | | | | |
|---|---|---|---|---|---|---|---|
| **HKG** | Hong Kong, China | 22.2095 | 114.2579 | 36.09 | 7.43 | 29.70 | 1.073 |
| **HKO** | King's Park (HKO,Hong Kong, China) | 22.312 | 114.173 | 33.55 | 6.57 | 26.18 | 1.063 |
| **HUN (10 m)** | Hegyhatsal (HUN (10 m), Hungary) | 46.95 | 16.65 | 13.13 | 2.07 | −1.92 | 0.994 |
| **HUN (48 m)** | Hegyhatsal (HUN (48 m), Hungary) | 46.95 | 16.65 | 8.38 | 1.48 | −1.48 | 0.997 |
| **HUN (82 m)** | Hegyhatsal (HUN (82 m), Hungary) | 46.95 | 16.65 | 7.24 | 1.31 | −1.31 | 0.997 |
| **HUN (115 m)** | Hegyhatsal (HUN (115 m), Hungary) | 46.965 | 16.65 | 6.73 | 1.23 | −1.23 | 0.997 |
| **LEF (396 m)** | Park Falls, Wisconsin, United States (LEF) | 45.93 | −90.27 | 3.89 | 0.73 | −0.29 | 0.999 |
| **ICE** | Storhofdi (ICE,Iceland) | 63.4 | −20.2833 | 0.97 | 0.57 | 1.39 | 1.003 |
| **ISK** | Issyk-Kul (ISK, Kyrgyzstan) | 42.61666 | 76.98333 | 10.99 | 2.43 | 8.89 | 1.023 |
| **KCO** | Kaashidhoo (Male Atoll) (KCO,Maldives) | 4.97 | 73.47 | 1.62 | 0.14 | −0.31 | 0.999 |
| **KEY** | Key Biscane (FL) (KEY,United States of America) | 25.66667 | −80.2 | 7.19 | 1.73 | 6.93 | 1.017 |
| **KOT** | Kotelnyj Island (KOT,Russian Federation) | 76 | 137.87 | 9.98 | 10.55 | 12.44 | 1.019 |
| **KPS** | K-Puszta (KPS,Hungary) | 46.96667 | 19.58333 | 7.11 | 7.38 | 8.32 | 0.997 |
| **KUM** | Cape Kumukahi (HI) (KUM,United States of America) | 19.52 | −154.82 | 1.70 | 0.37 | 0.89 | 1.002 |
| **KYZ** | Kyzylcha (KYZ,Uzbekistan) | 40.87 | 66.15 | 2.50 | 6.99 | 7.12 | 1.016 |
| **KZD** | Sary Taukum (KZD,Kazakhstan) | 44.45 | 77.57 | 8.84 | 2.15 | 8.28 | 1.021 |
| **KZM** | Plateau Assy (KZM,Kazakhstan) | 43.25 | 77.88 | 11.60 | 2.93 | 11.19 | 1.029 |
| **LEF** | Park Falls (WI) (LEF,United States of America) | 45.93 | −90.27 | 6.94 | 1.95 | 3.93 | 1.021 |
| **LLN** | Lulin (LLN,Taiwan, Province of China) | 23.47 | 120.87 | 29.67 | 7.35 | 29.07 | 1.073 |
| **LPO** | Ile Grande (LPO,France) | 48.8036 | −3.5839 | 3.97 | 0.93 | 3.48 | 1.009 |
| **MAA** | Mawson (MAA,Australia) | −67.6047 | 62.87056 | 1.27 | 0.29 | 0.18 | 1 |
| **MDN** | Madonie - Piano Battaglia (MDN,Italy) | 37.88031 | 14.02564 | 12.39 | 2.97 | 11.27 | 1.028 |
| **MID** | Sand Island (MID,United States of America) | 28.22 | −177.37 | 1.95 | 0.41 | 1.01 | 1.003 |
| **MKN** | Mt. Kenya (MKN,Kenya) | −0.0622 | 37.2972 | 8.62 | 2.14 | 8.17 | 1.021 |
| **NAT** | Natal (NAT,Brazil) | −6 | −35.2 | 1.64 | 0.33 | 1.04 | 1.003 |
| **NMB** | Gobabeb (NMB,Namibia) | −23.57 | 15.03 | 1.19 | 0.24 | −0.51 | 0.999 |
| **NWR** | Niwot Ridge - T-van (CO) (NWR,United States of America) | 40.05 | −105.59 | 17.63 | 4.19 | 16.50 | 1.042 |
| **OXK** | Ochsenkopf (OXK,Germany) | 50.0301 | 11.8084 | 10.38 | 2.42 | 9.58 | 1.024 |
| **PDM** | Pic du Midi (PDM,France) | 42.9372 | 0.1411 | 13.17 | 3.27 | 12.57 | 1.033 |
| **PSA** | Palmer Station (PSA,United States of America) | −64.7743 | −64.0544 | 1.32 | 0.30 | 0.27 | 1.001 |
| **PTA** | Point Arena (CA) (PTA,United States of America) | 38.95 | −123.73 | 7.03 | 1.54 | 5.68 | 1.015 |

| | | | | | | | |
|---|---|---|---|---|---|---|---|
| **RGL (45 m)** | Ridge Hill (RGL,United Kingdom of Great Britain and Northern Ireland) | 51.99756 | −2.54003 | 3.50 | 0.61 | −1.54 | 0.996 |
| **RGL (90 m)** | Ridge Hill (RGL,United Kingdom of Great Britain and Northern Ireland) | 51.99756 | −2.54003 | 3.39 | 0.59 | −1.44 | 0.996 |
| **RPB** | Ragged Point (RPB,Barbados) | 13.17 | −59.43 | 1.83 | 0.39 | 1.13 | 1.003 |
| **SDZ** | Shangdianzi (SDZ,China) | 40.65 | 117.1166 | 23.06 | 5.41 | 21.55 | 1.053 |
| **SEY** | Mahé (SEY,Seychelles) | −4.67 | 55.17 | 1.92 | 0.51 | 1.92 | 1.001 |
| **SGP** | Southern Great Plains E13 (OK) (SGP,United States of America) | 36.6 | −97.5 | 13.75 | 3.61 | 13.75 | 1.02 |
| **SHM** | Shemya Island (SHM,United States of America) | 52.72 | 174.1 | 2.66 | 0.51 | 0.87 | 1.002 |
| **STM** | Ocean Station M (STM,Norway) | 66 | 2 | 2.34 | 0.53 | 1.98 | 1.005 |
| **SUM** | Summit (SUM,Denmark) | 72.58 | −38.48 | 1.95 | 0.43 | 1.03 | 1.003 |
| **SYO** | Syowa (SYO,Japan) | −69.0053 | 39.5811 | 1.36 | 0.31 | 0.44 | 1.001 |
| **TAC (54 m)** | Tacolneston Tall Tower (TAC (54 m),United Kingdom of Great Britain and Northern Ireland) | 52.5177 | 1.1386 | 3.95 | 0.70 | −1.59 | 0.996 |
| **TAC (100 m)** | Tacolneston Tall Tower (TAC (100 m),United Kingdom of Great Britain and Northern Ireland) | 52.5177 | 1.1386 | 9.20 | 1.81 | −0.97 | 0.997 |
| **TAC (185 m)** | Tacolneston Tall Tower (TAC (185 m),United Kingdom of Great Britain and Northern Ireland) | 52.5177 | 1.1386 | 2.99 | 0.54 | −0.27 | 0.999 |
| **TAP** | Tae-ahn Peninsula (TAP,Republic of Korea) | 36.73 | 126.13 | 17.45 | 4.17 | 16.75 | 1.042 |
| **TER** | Teriberka (TER,Russian Federation) | 69.2 | 35.1 | 2.62 | 0.54 | 1.10 | 1.003 |
| **THD** | Trinidad Head (CA) (THD,United States of America) | 41.0541 | −124.151 | 8.27 | 1.79 | 6.98 | 1.018 |
| **USH** | Ushuaia (USH,Argentina) | −54.8485 | −68.3107 | 1.49 | 0.34 | 0.72 | 1.002 |
| **UTA** | Wendover (UT) (UTA,United States of America) | 39.9 | −113.72 | 3.09 | 0.65 | 2.46 | 1.006 |
| **UUM** | Ulaan Uul (UUM,Mongolia) | 44.444 | 111.0861 | 5.55 | 1.16 | 4.34 | 1.011 |
| **WIS** | Sede Boker (WIS,Israel) | 31.13 | 34.88 | 6.47 | 1.55 | 6.09 | 1.015 |
| **WLG** | Mt. Waliguan (WLG,China) | 36.2875 | 100.8963 | 5.52 | 1.13 | 4.59 | 1.011 |
| **WBI 31m** | West Branch, Iowa, United States (WBI 31m) | 41.7248 | −91.3529 | 10.11 | 1.75 | −3.46 | 0.992 |
| **WBI 99m** | West Branch, Iowa, United States (WBI 99m) | 41.7248 | −91.3529 | 7.79 | 1.40 | −2.23 | 0.994 |
| **WBI 379m** | West Branch, Iowa, United States (WBI 379m) | 41.7248 | −91.3529 | 6.54 | 1.19 | −0.74 | 0.997 |
| **WKT 9m** | Moody, Texas, United States (WKT 9m) | 31.3149 | −97.3269 | 16.39 | 2.86 | 6.15 | 1.015 |

| | | | | | | | |
|---|---|---|---|---|---|---|---|
| **WKT** **30m** | Moody, Texas, United States (WKT 30m) | 31.3149 | −97.3269 | 12.73 | 2.41 | 5.77 | 1.015 |
| **WKT** **61m** | Moody, Texas, United States (WKT 61m) | 31.3149 | −97.3269 | 10.82 | 2.07 | 3.16 | 1.008 |
| **WKT** **122m** | Moody, Texas, United States (WKT 122m) | 31.3149 | −97.3269 | 8.63 | 1.73 | 1.61 | 1.004 |
| **WKT** **244m** | Moody, Texas, United States (WKT 244m) | 31.3149 | −97.3269 | 7.58 | 1.55 | 3.46 | 1.009 |
| **WKT** **457m** | Moody, Texas, United States (WKT 457m) | 31.3149 | −97.3269 | 6.57 | 1.34 | 2.83 | 1.007 |
| **SCT** **31m** | Beech Island, South Carolina, United States (SCT 31m) | 33.4057 | −81.8334 | 13.99 | 2.21 | 7.21 | 1.017 |
| **SCT** **61m** | Beech Island, South Carolina, United States (SCT 61m) | 33.4057 | −81.8334 | 10.71 | 1.82 | 6.03 | 1.014 |
| **SCT** **305m** | Beech Island, South Carolina, United States (SCT 305m) | 33.4057 | −81.8334 | 5.99 | 1.09 | 3.37 | 1.008 |
